# Nocodazole-Induced Expression and Phosphorylation of Anillin and Other Mitotic Proteins Are Decreased in DNA-Dependent Protein Kinase Catalytic Subunit-Deficient Cells and Rescued by Inhibition of the Anaphase-Promoting Complex/Cyclosome with proTAME but Not Apcin

**DOI:** 10.1128/MCB.00191-19

**Published:** 2020-06-15

**Authors:** Pauline Douglas, Ruiqiong Ye, Suraj Radhamani, Alexander Cobban, Nicole P. Jenkins, Edward Bartlett, Jonathan Roveredo, Arminja N. Kettenbach, Susan P. Lees-Miller

**Affiliations:** a Department of Biochemistry and Molecular Biology and Robson DNA Science Centre, Cumming School of Medicine, University of Calgary, Calgary, Alberta, Canada; b Norris Cotton Cancer Center, Geisel School of Medicine, Lebanon Campus at Dartmouth-Hitchcock Medical Center, Lebanon, New Hampshire, USA

**Keywords:** APC/C, Aurora A, DNA-PK, PLK1, TPX2, anillin, mitosis, nocodazole, protein kinase

## Abstract

The DNA-dependent protein kinase catalytic subunit (DNA-PKcs) has well-established roles in DNA double-strand break repair, and recently, nonrepair functions have also been reported. To better understand its cellular functions, we deleted DNA-PKcs from HeLa and A549 cells using CRISPR/Cas9. The resulting cells were radiation sensitive, had reduced expression of ataxia-telangiectasia mutated (ATM), and exhibited multiple mitotic defects. Mechanistically, nocodazole-induced upregulation of cyclin B1, anillin, and securin was decreased in DNA-PKcs-deficient cells, as were phosphorylation of Aurora A on threonine 288, phosphorylation of Polo-like kinase 1 (PLK1) on threonine 210, and phosphorylation of targeting protein for *Xenopus* Klp2 (TPX2) on serine 121. Moreover, reduced nocodazole-induced expression of anillin, securin, and cyclin B1 and phosphorylation of PLK1, Aurora A, and TPX2 were rescued by inhibition of the anaphase-promoting complex/cyclosome (APC/C) by proTAME, which prevents binding of the APC/C-activating proteins Cdc20 and Cdh1 to the APC/C. Altogether, our studies suggest that loss of DNA-PKcs prevents inactivation of the APC/C in nocodazole-treated cells.

## Abstract (precis)

The DNA-dependent protein kinase catalytic subunit (DNA-PKcs) has well-established roles in DNA double-strand break repair, and recently, nonrepair functions have also been reported. To better understand its cellular functions, we deleted DNA-PKcs from HeLa and A549 cells using CRISPR/Cas9. The resulting cells were radiation sensitive, had reduced expression of ataxia-telangiectasia mutated (ATM), and exhibited multiple mitotic defects. Mechanistically, nocodazole-induced upregulation of cyclin B1, anillin, and securin was decreased in DNA-PKcs-deficient cells, as were phosphorylation of Aurora A on threonine 288, phosphorylation of Polo-like kinase 1 (PLK1) on threonine 210, and phosphorylation of targeting protein for *Xenopus* Klp2 (TPX2) on serine 121.

## INTRODUCTION

The DNA-dependent protein kinase catalytic subunit (DNA-PKcs/*PRKDC*) is a member of the phosphatidylinositol-3 kinase-like protein kinase (PIKK) family of serine/threonine protein kinases (1, 2). DNA-PKcs has well-established roles in nonhomologous end joining (NHEJ), the major pathway for repair of ionizing-radiation-induced DNA double-strand breaks (DSBs) in human cells, where it interacts with the Ku70/Ku80 heterodimer and DSB ends to regulate NHEJ. DNA-PKcs, with the nuclease Artemis, is also required for opening DNA hairpins ends in V(D)J recombination in the adaptive immune system (3, 4). Recently, increasing evidence suggests that DNA-PKcs also has multiple roles outside DNA repair. For example, DNA-PKcs localizes to the promoters of nuclear receptor genes to regulate transcription (5–7) and interacts with the androgen receptor to regulate cell invasion and metastasis in prostate cancer (6). In addition, DNA-PKcs has been reported to regulate protein secretion (8), the cytoskeleton (9), and innate immunity (10). Recently, additional roles in regulation of metabolism (11), cellular senescence (12), protein synthesis, and ribosome processing (13, 14) have also been described. In addition, we and others have shown that loss or inhibition of DNA-PKcs results in multiple defects in mitosis (12, 15–20) and cytokinesis (12) in human cells.

DNA-PKcs is autophosphorylated in mitosis (15, 16), localizes to the mitotic spindle, centrosomes, and midbody, interacts with mitotic Polo-like kinase 1 (PLK1) (16, 17, 19), and is phosphorylated by PLK1 in mitotic human cells (16). DNA-PKcs-depleted cells treated with the microtubule-depolymerizing agent nocodazole (21) have elevated levels of tyrosine 15-phosphorylated cyclin-dependent kinase 1 (CDK1), elevated expression of Myt1 and Wee1 protein kinases, and decreased CDC25 phosphorylation, consistent with delayed and/or decreased activation of CDK1 and, consequently, delayed or attenuated mitotic entry (19). In addition, small interfering RNA (siRNA) depletion of DNA-PKcs and/or inhibition of its catalytic activity results in chromosome misalignment and prolonged passage through mitosis in either nocodazole-treated cells, cells undergoing normal cellular progression (15, 16, 21), or cells exposed to ionizing radiation (22). Moreover, DNA-PKcs-depleted cells display chromosome segregation defects and cytokinesis defects in both irradiated (17) and unirradiated (12) cells and elevated levels of cyclin B1 postanaphase, blocking mitotic exit (18).

To better understand its multiple cellular functions, we deleted DNA-PKcs from human cells using CRISPR/Cas9 and determined the effects of DNA-PKcs deletion on molecular markers of mitotic entry. Nocodazole-induced expression of cyclin B1, securin. and anillin was dramatically reduced in DNA-PKcs-depleted cells, as was phosphorylation of PLK1, Aurora A, and targeting protein of *Xenopus* Klp2 (TPX2). Moreover, reduced anillin, securin, and cyclin B1 levels in nocodazole-treated DNA-PKcs-deficient cells were rescued by inhibition of the anaphase-promoting complex/cyclosome (APC/C) with the cell-permeable small molecule proTAME (Pro-*N*-4-tosyl-l-arginine methyl ester), which disrupts loading of Cdc20 and Cdh1 onto the APC/C, but not with apcin, which disrupts interaction of Cdc20 and Cdh1 with APC/C target proteins (23, 24). Together, our results suggest that loss of DNA-PKcs prevents activation of the spindle assembly checkpoint (SAC) and/or inactivation of the APC/C, causing inappropriate degradation of mitotic proteins, which in turn contributes to the multiple mitotic defects observed in DNA-PKcs-deficient cells.

## RESULTS

### Nocodazole-treated DNA-PKcs-depleted human cells have reduced expression and/or phosphorylation of multiple proteins required for mitotic entry, progression, exit, and cytokinesis.

**FIG 1:**
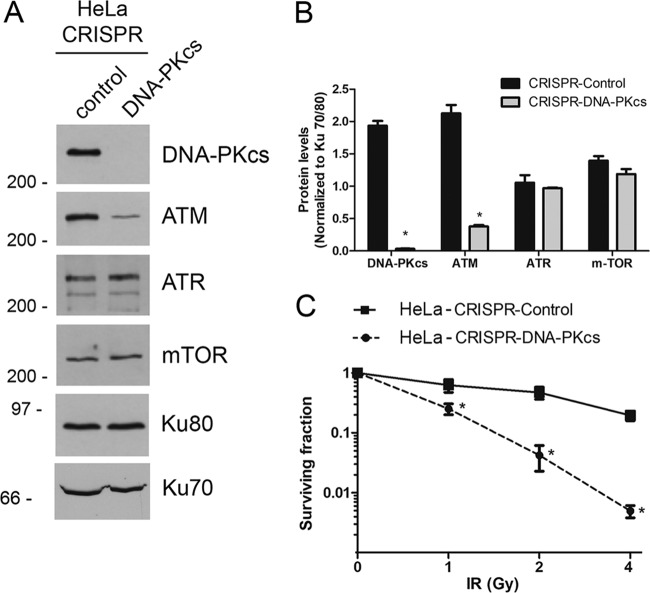
HeLa–CRISPR–DNA-PKcs cells have reduced levels of ATM and are sensitive to ionizing radiation (IR). (A) HeLa-CRISPR-control and HeLa–CRISPR–DNA-PKcs cells were harvested with trypsin EDTA and lysed in NETN buffer containing protease and phosphatase inhibitors (40). Fifty micrograms of total protein was run on SDS-PAGE and immunoblotted for DNA-PKcs, ATM, ATR, mTOR, Ku70, and Ku80 (loading control) as shown on the right. (B) Quantification from three separate experiments. Statistical significance was determined by one-way ANOVA. *, *P* < 0.05. (C) HeLa-CRISPR-control and HeLa–CRISPR–DNA-PKcs cells were either not irradiated (0) or irradiated with 1, 2, or 4 Gy of IR, and radiation sensitivity was determined using a clonogenic survival assay. The results represent the average number of colonies from three separate experiments. Statistical significance was determined by one-way ANOVA (*, *P* < 0.05). The error bars indicate standard deviations.

To better understand the functions of DNA-PKcs, we used CRISPR/Cas9 to delete DNA-PKcs from HeLa cells, a human cell line derived from human papillomavirus 16 (HPV-16)-positive cervical carcinoma. As expected, no DNA-PKcs protein was detected by Western blotting in the CRISPR–DNA-PKcs cells (Fig. 1A and B) and, in keeping with previous studies in the DNA-PKcs-null human cell line M059J (25, 26) and in mammalian cells treated with siRNA to DNA-PKcs (6, 8, 27), cells with CRISPR/Cas9 deletion of DNA-PKcs had an approximately 80% reduction in expression of the related protein kinase, ataxia telangiectasia mutated (ATM). In contrast, levels of other PIKKs such as ATM and Rad3-related (ATR) and mammalian/mechanistic target of rapamycin (mTOR) were unaffected by DNA-PKcs loss (Fig. 1A and B). As expected from previous studies in rodent (28) and human (26) cells, DNA-PKcs-deficient cells were sensitive to ionizing radiation (IR) (Fig. 1C).

We next examined the cell cycle kinetics and morphology of the DNA-PKcs-deficient cells using live-cell imaging. Consistent with previous reports, HeLa cells with DNA-PKcs deleted took longer to pass from prophase to anaphase (15) and exhibited multiple abnormalities during cytokinesis, including membrane blebbing and cell fusions (12, 17, 22; data not shown). To investigate the molecular mechanisms responsible for these profound mitotic defects, HeLa-CRISPR-control and HeLa–CRISPR–DNA-PKcs cells were incubated with nocodazole, a microtubule inhibitor that disrupts attachment of kinetochores to microtubules, leading to activation of the SAC, inhibition of the APC/C, and arrest in prometaphase (21). As reported previously for HeLa cells with siRNA depletion of DNA-PKcs (19), HeLa–CRISPR–DNA-PKcs cells had a reduced mitotic index (percentage of cells staining positive for the mitotic markers histone H3pS10 [29] and MPM2 [30] after nocodazole treatment [data not shown]). Also, as seen with siRNA depletion of DNA-PKcs, incubation of HeLa–CRISPR–DNA-PKcs cells with nocodazole led to an increased percentage of cells with polylobed nuclei, micronuclei, and chromosome bridges, indicators of genome instability (15, 16; data not shown).

**FIG 2:**
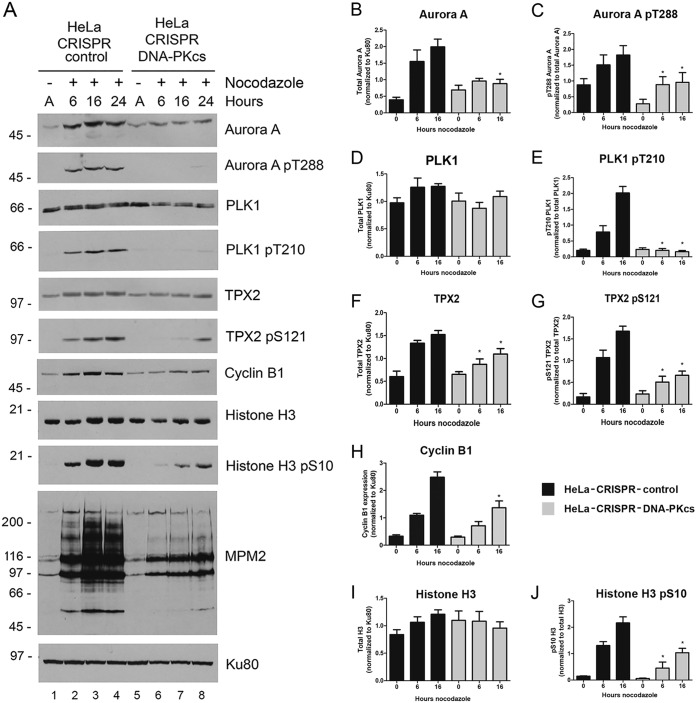
Phosphorylation of Aurora A and PLK1 and accumulation of cyclin B1 are delayed and/or reduced in nocodazole-treated HeLa–CRISPR–DNA-PKcs cells. (A) HeLa-CRISPR-control and HeLa–CRISPR–DNA-PKcs cells were grown under asynchronous conditions (lane A) or incubated with nocodazole (40 ng/ml) for 6, 16, or 24 h, as indicated, harvested, and analyzed by SDS-PAGE and immunoblotted for markers of mitosis, as indicated on the right. The blots were probed for Ku80 as a loading control. The data are representative of at least 3 separate experiments. (B to J) Quantitation for HeLa-CRISPR-control cells and HeLa–CRISPR–DNA-PKcs cells. Total proteins were normalized to Ku80 as a loading control. Phosphoproteins were normalized to their respective total proteins. Results are shown in arbitrary units on the *y* axis. Statistical significance was determined using Student's *t* test. *P* values of <0.05 (*) were taken as statistically significant. The error bars indicate standard deviations.

To better understand the mechanism of the nocodazole-induced mitotic defects and genome instability observed in DNA-PKcs-deficient cells, we examined the effects of DNA-PKcs loss on nocodazole-induced expression of cyclin B1 and additional markers of mitotic entry. Specifically, we probed for expression of Aurora A and autophosphorylation on its activation loop site, threonine 288, expression of PLK-1 and phosphorylation of the activation loop site threonine 210 (31, 32) and TPX2, a microtubule-associated protein required for spindle assembly and activation of Aurora A, which is phosphorylated by Aurora A on serine 121 (33). HeLa-CRISPR-control and HeLa–CRISPR–DNA-PKcs cells were incubated with nocodazole for 6, 16, or 24 h, and expression of mitotic markers was analyzed by Western blotting. As reported for cells with siRNA-mediated depletion of DNA-PKcs (19), nocodazole-induced PLK1 T210 phosphorylation was decreased in HeLa–CRISPR–DNA-PKcs cells (Fig. 2A and E), as was phosphorylation of Aurora A on T288 (Fig. 2A and C). In addition, expression of Aurora A protein, but not total PLK1 protein, was decreased in nocodazole-treated cells with DNA-PKcs deleted (Fig. 2A, B, and D). Similarly, expression of TPX2 protein and phosphorylation on S121 phosphorylation were reduced (Fig. 2A, F, and G), as was phosphorylation of histone H3-S10 (Fig. 2A, I, and J). Thus, loss of DNA-PKcs results in reduced nocodazole-induced phosphorylation of PLK1, Aurora A, TPX2, and histone H3, known markers of mitotic entry (31, 32). In addition, nocodazole-induced cyclin B1 protein expression was decreased in DNA-PKcs-deficient cells (Fig. 2A and H). Thus, loss of DNA-PKcs reduces both accumulation of cyclin B1 in the presence of nocodazole (this study) and degradation of cyclin B1 after release from nocodazole and exit from mitosis (18, 19).

Decreased upregulation of cyclin B1 would also be consistent with reduced CDK1 activity at G_2_/M, as proposed previously (19). As a surrogate for CDK1 activity, we probed immunoblots with MPM2, a monoclonal antibody that recognizes multiple proteins phosphorylated at serine-proline and threonine-proline sites, i.e., CDK1 sites, at mitotic entry (30, 34). MPM2 antibody signal was decreased in nocodazole-treated DNA-PKcs-deficient cells, again consistent with loss of DNA-PKcs having a profound effect upon activation of CDK1 and phosphorylation of mitotic proteins at mitotic entry (Fig. 2A).

**FIG 3:**
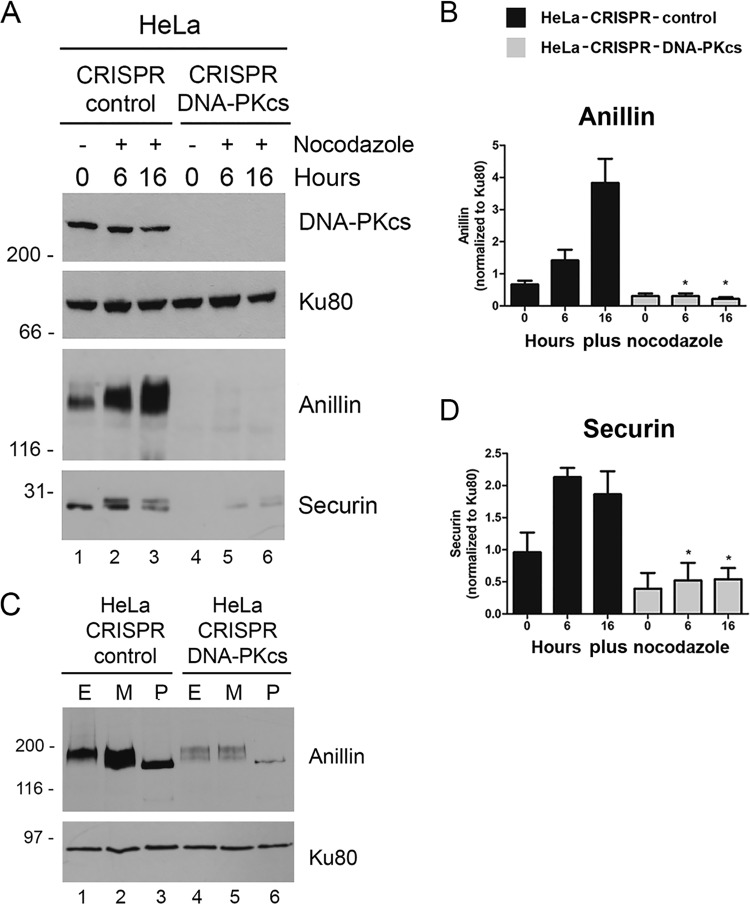
Nocodazole-induced expression of anillin and securin is reduced in HeLa–CRISPR–DNA-PKcs cells. (A) HeLa-CRISPR-control and HeLa–CRISPR–DNA-PKcs cells were incubated in the absence (0) or presence of nocodazole (40 ng/ml) and harvested after 6 or 16 h, as indicated. Whole-cell extracts were generated, run on SDS-PAGE, and immunoblotted as for Fig. 2. (B and D) Quantitation of anillin and securin expression from three separate experiments as for Fig. 2. Statistical analysis was carried out as for Fig. 2. (C) Whole-cell extracts (lanes E) from nocodazole-treated (40 ng/ml; 16 h) HeLa-CRISPR-control (lanes 1 to 3) or HeLa–CRISPR–DNA-PKcs (lanes 4 to 6) cells were run on SDS-PAGE either directly (lanes E) or after incubation with 0.5 μl lambda phosphatase at 30°C for 10 min (lanes P) or incubated without phosphatase (mock treated; lanes M). Extracts were run on SDS-PAGE and immunoblotted for anillin and Ku80, as shown. The error bars indicate standard deviations.

To examine whether loss of DNA-PKcs also affected markers of mitotic exit and cytokinesis, we examined expression of anillin and securin in nocodazole-treated cells. Anillin is an actin-binding protein that is required for cytokinesis (35), while securin is a regulator of separase, a protease required for chromosome segregation (36). Consistent with previous findings (37), anillin was present at low levels in asynchronously growing cells, and its levels increased beginning 6 h after exposure to nocodazole (Fig. 3A and B). The anillin-cross-reacting bands also migrated more slowly after nocodazole treatment, consistent with enhanced phosphorylation (Fig. 3A). Indeed, treatment of extracts with lambda phosphatase caused the anillin-cross-reacting bands to collapse into one faster-migrating band, indicating that the more slowly migrating bands were indeed due to phosphorylation (Fig. 3C). Moreover, both expression and phosphorylation of anillin were significantly decreased in nocodazole-treated HeLa–CRISPR–DNA-PKcs cells (Fig. 3A to C). Similarly, expression of securin was greatly reduced in nocodazole-treated HeLa–CRISPR–DNA-PKcs cells (Fig. 3A and D). Thus, loss of DNA-PKcs reduces the nocodazole-induced phosphorylation and/or expression of proteins required for mitotic entry (cyclin B1, Aurora A, TPX2, and PLK1), as well as mitotic exit and cytokinesis (anillin, securin, and cyclin B1). We note that upregulation/phosphorylation of these mitotic proteins is not absent, as suggested by the experiments shown in Fig. 3A; rather, phosphorylation/upregulation is decreased compared to control cells, as in other experiments and/or in darker exposures of the experiment shown in Fig. 3A, anillin protein was detected, just at much lower levels than in control cells (Fig. 3C).

A caveat of previously published data using short hairpin RNA (shRNA) to DNA-PKcs (18, 22), as well the data described above, is that the depletion of DNA-PKcs leads, through an as yet unidentified mechanism, to reduced expression of the related protein kinase ATM (Fig. 1A). It was therefore important to separate effects due to loss of DNA-PKcs with correspondingly low levels of ATM from those due to loss of DNA-PKcs alone. We also wanted to determine whether loss of DNA-PKcs had similar effects on mitotic proteins in a cell line without HPV-associated loss of p53 function. To this end, we deleted DNA-PKcs and ATM from the lung adenocarcinoma cell line A549, which expresses functional p53 protein (38). To avoid potential bias due to off-target effects, different guide RNAs than those used for DNA-PKcs depletion in HeLa cells (this study) were used for depletion of DNA-PKcs in A549 cells (39). By Western blotting, we found that ATM protein levels were again low in A549–CRISPR–DNA-PKcs cells but that A549-CRISPR-ATM cells had apparently normal levels of DNA-PKcs. As expected, both A549–CRISPR–DNA-PKcs and A549-CRISPR-ATM cell lines were sensitive to IR (39).

**FIG 4:**
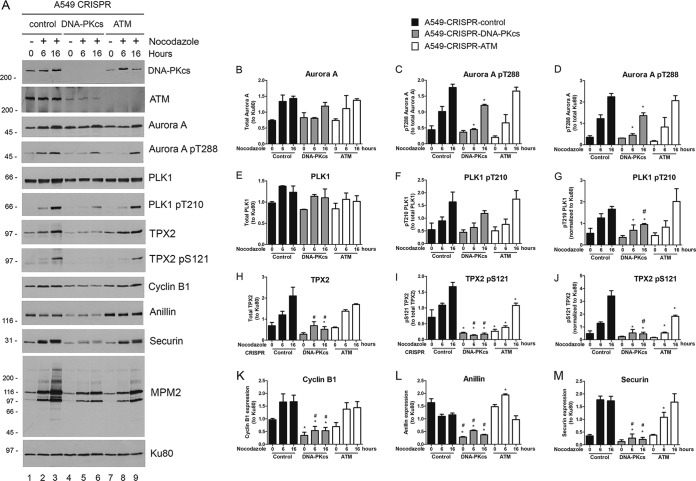
A549 cells with CRISPR depletion of DNA-PKcs have reduced and/or delayed nocodazole-induced upregulation of anillin, cyclin B1, and securin and reduced phosphorylation of Aurora A, PLK1, and TPX2. (A) A549-CRISPR-control, A549–CRISPR–DNA-PKcs, and A549-CRISPR-ATM cells were treated with nocodazole for 6 or 16 h, and then extracts were run on SDS-PAGE and analyzed by Western blotting as for Fig. 2. (B to M) Quantitation from 3 separate experiments. (B, D, E, G, H, J, K, L, and M) Proteins were normalized to Ku80. (C, F, and I) Phosphoproteins were normalized to their respective total proteins. Both are shown in arbitrary units (*y* axis). Statistical analysis was carried out by one-way ANOVA, with *P* values of <0.05 considered statistically significant. *, statistically significant difference between control and CRISPR-DNA-PKcs cells; #, statistical significance between CRISPR-DNA-PKcs and CRISPR-ATM cells. The error bars indicate standard deviations.

We next examined the expression of cyclin B1, anillin, and securin and phosphorylation of Aurora A, PLK1, TPX2, and MPM2-cross-reacting phosphoproteins in the panel of A549 cells. As seen in HeLa cells, nocodazole-induced expression of anillin, securin, and cyclin B1 was reduced in A549–CRISPR–DNA-PKcs cells, as was phosphorylation of Aurora A and phosphorylation of mitotic proteins recognized by the MPM2 antibody (Fig. 4A). Moreover, phosphorylation/upregulation of anillin, securin, cyclin B1, and TPX2 was decreased to a greater extent in A549–CRISPR–DNA-PKcs cells than in A549-CRISPR-ATM cells (Fig. 4A and F to J), again, strongly implying a role for DNA-PKcs in nocodazole-induced upregulation of mitotic proteins. However, we noted similarity in phosphorylation of MPM2-cross-reacting proteins between nocodazole-treated CRISPR-DNA-PKcs and CRISPR-ATM cells (Fig. 4A), suggesting that ATM also plays a role in phosphorylation of some mitotic proteins in nocodazole-treated cells. Together, these results indicate that while both DNA-PKcs and ATM may contribute to phosphorylation of many mitotic proteins in response to nocodazole, some, in particular phosphorylation and upregulation of TPX2, anillin, and securin, appear to be more DNA-PK dependent than ATM dependent.

### Nocodazole-induced upregulation/phosphorylation of mitotic proteins does not require PP6.

**FIG 5:**
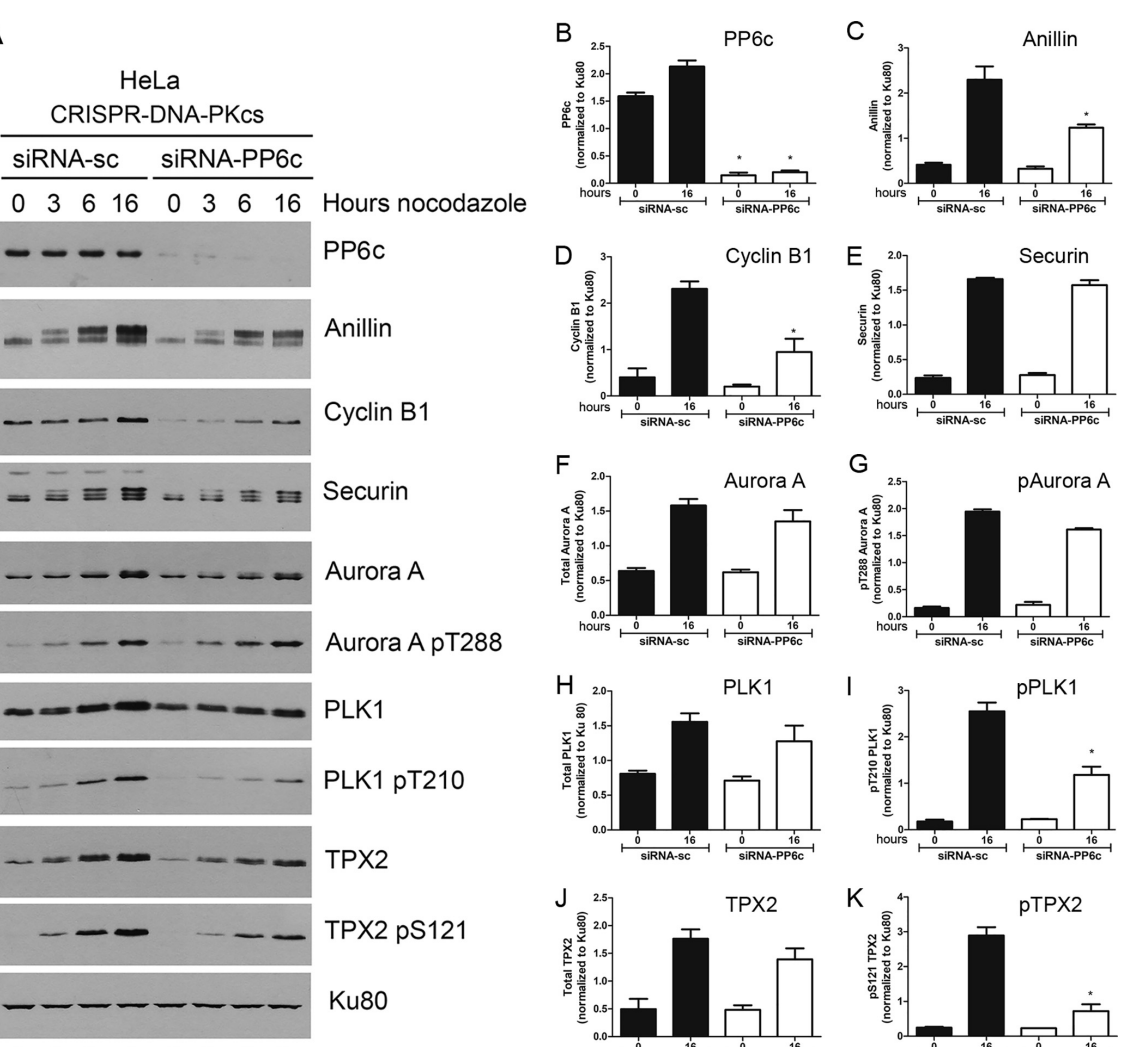
Depletion of PP6 does not rescue nocodazole-induced downregulation of mitotic proteins in DNA-PKcs-deficient HeLa cells. (A) HeLa–CRISPR–DNA-PKcs cells were transfected with either scrambled control siRNA (siRNA-sc) or siRNA to PP6c (siRNA-PP6c) (100 nM), and after 56 h, the cells were treated with nocodazole (40 ng/ml) and harvested immediately (0) or after 3, 6, or 16 h, as indicated. Extracts were probed for mitotic proteins as described above. The Western blots are representative of 3 separate experiments. (B to K) Quantitation shown as in Fig. 2 and 4. The asterisk on the securin Western blot indicates a nonspecific band. siRNA-sc, scrambled siRNA control. The error bars indicate standard deviations.

**FIG 6:**
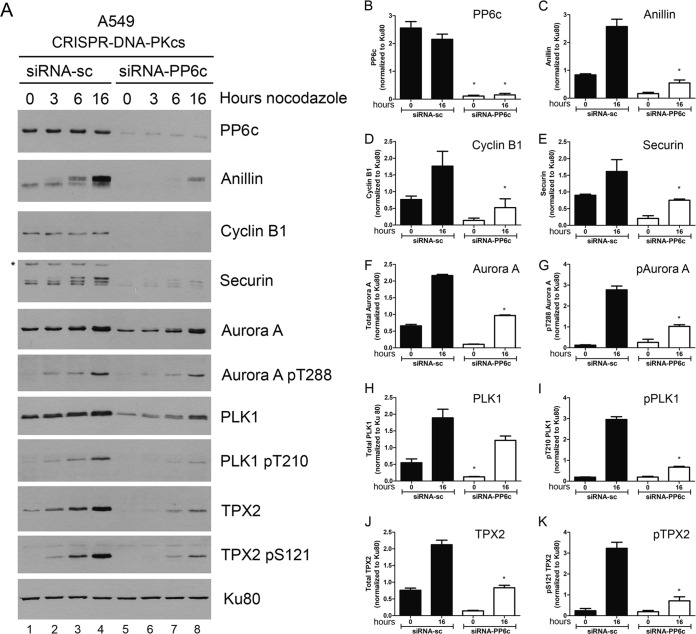
Depletion of PP6 does not rescue nocodazole-induced downregulation of mitotic proteins in DNA-PKcs-deficient A549 cells. The experiment was carried out exactly as for Fig. 5, but A549–CRISPR–DNA-PKcs cells were used. The asterisk on the securin blot indicates a nonspecific band. The error bars indicate standard deviations.

DNA-PKcs interacts with protein phosphatase 6 (PP6) (40, 41), and PP6 has been shown to regulate spindle formation and Aurora A activity (42) and to have multiple roles in mitosis (43). We therefore hypothesized that loss of DNA-PKcs might lead to upregulation and/or activation of PP6 activity, leading to decreased phosphorylation of mitotic proteins, such as anillin, securin, and TPX2, which, in addition, could regulate protein expression levels. If this hypothesis was correct, then depletion of PP6 in CRISPR-DNA-PKcs cells would be expected to rescue the defects in nocodazole-induced phosphorylation and/or upregulation of anillin and other mitotic proteins observed in DNA-PKcs-deficient cells. To test this hypothesis, we depleted the PP6 catalytic subunit (PP6c) from CRISPR-DNA-PKcs cells and then treated them with nocodazole as described above. Note that in these experiments, immunoblots were exposed for longer times to visualize proteins in the CRISPR-DNA-PKcs cells. Deletion of PP6c did not rescue nocodazole-induced expression or phosphorylation of any of the mitotic proteins tested; indeed, in most cases, phosphorylation of mitotic proteins in nocodazole-treated CRISPR-DNA-PKcs cells was decreased (Fig. 5 and 6). Thus, we conclude that DNA-PKcs is unlikely to regulate phosphorylation of mitotic proteins through downregulation of PP6.

### Inhibition of the APC/C rescues decreased phosphorylation and expression of mitotic proteins in nocodazole-treated DNA-PKcs-deficient cells.

We next asked whether DNA-PKcs-dependent regulation of expression and phosphorylation of mitotic proteins could be occurring through activation of the SAC. Nocodazole is a microtubule-depolymerizing agent that prevents attachment of kinetochores to microtubules. This, in turn, activates the SAC, preventing the APC/C from targeting mitotic proteins for degradation by the proteasome (44, 45). Indeed, cyclin B1, securin, and anillin are known to be APC/C targets (37, 46, 47). Critical to APC/C activation is the mitotic checkpoint complex (MCC), composed of Mad2, Bub3, BubR1, and Cdc20 (48) and the APC/C-activating proteins Cdc20 and Cdh1/Fzr1 (49). When the SAC is not satisfied (for example, in nocodazole-treated cells with unattached kinetochores), Cdc20 is sequestered by the MCC, APC/C is inactive, and levels of APC/C target proteins, such as anillin, cyclin B1, and securin, rise (48). When the SAC is satisfied, Cdc20 is released, cyclin B, securin, and other proteins are recruited to the APC/C by Cdc20 or Cdh1, the APC/C is activated, and the target proteins are degraded so that the cell can proceed into anaphase and exit mitosis (48). Thus, we hypothesized that loss of DNA-PKcs might prevent activation of the SAC and/or inactivation of the APC/C, allowing cyclin B1, securin, and anillin to be degraded by the proteasome, even in the presence of unattached kinetochores.

To test this hypothesis, we examined the effects of two small-molecule APC/C inhibitors, apcin and proTAME (23), on anillin, securin, and cyclin B1 expression and Aurora A, PLK1, and TPX2 phosphorylation in nocodazole-treated CRISPR-DNA-PKcs cells. Proteins that interact with Cdc20 or Cdh1 do so via specific amino acid motifs, such as KEN boxes and destruction (D) boxes (47). Apcin binds to the D-box-binding pocket of Cdc20, preventing it from interacting with and activating the APC/C, while proTAME prevents Cdc20 and Cdh1 from loading onto the APC/C (49). Thus, if loss of DNA-PKcs resulted in inability to inactivate the SAC, inhibition of the APC/C by apcin and/or proTAME would be expected to rescue the levels of anillin, securin, and cyclin B1 in nocodazole-treated CRISPR-DNA-PKcs cells.

**FIG 7:**
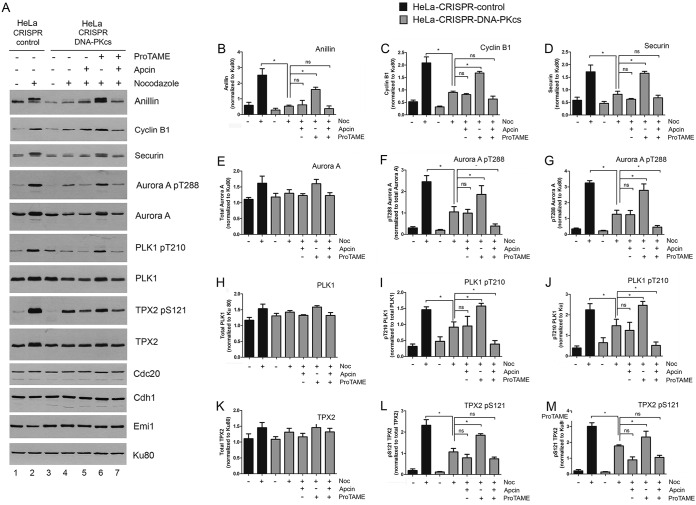
Inhibition of APC/C with proTAME rescues expression and phosphorylation of mitotic proteins in nocodazole-treated DNA-PKcs-deficient HeLa cells. HeLa-CRISPR-control and HeLa–CRISPR–DNA-PKcs cells were preincubated with apcin (48 μM), proTAME (12 μM), or DMSO control for 1 h, and then nocodazole was added to 40 ng/ml and cells were harvested after a further 16 h. Representative Western blots (A) and quantitation from 3 separate experiments (B to M) are shown as in Fig. 5 and 6. (A, B, C, D, E, G, H, J, K, and M) Proteins were normalized to Ku80. (F, I, and L) Phosphoproteins were normalized to their respective total protein controls. The error bars indicate standard deviations. *, *P* < 0.05; ns, not significant.

**FIG 8:**
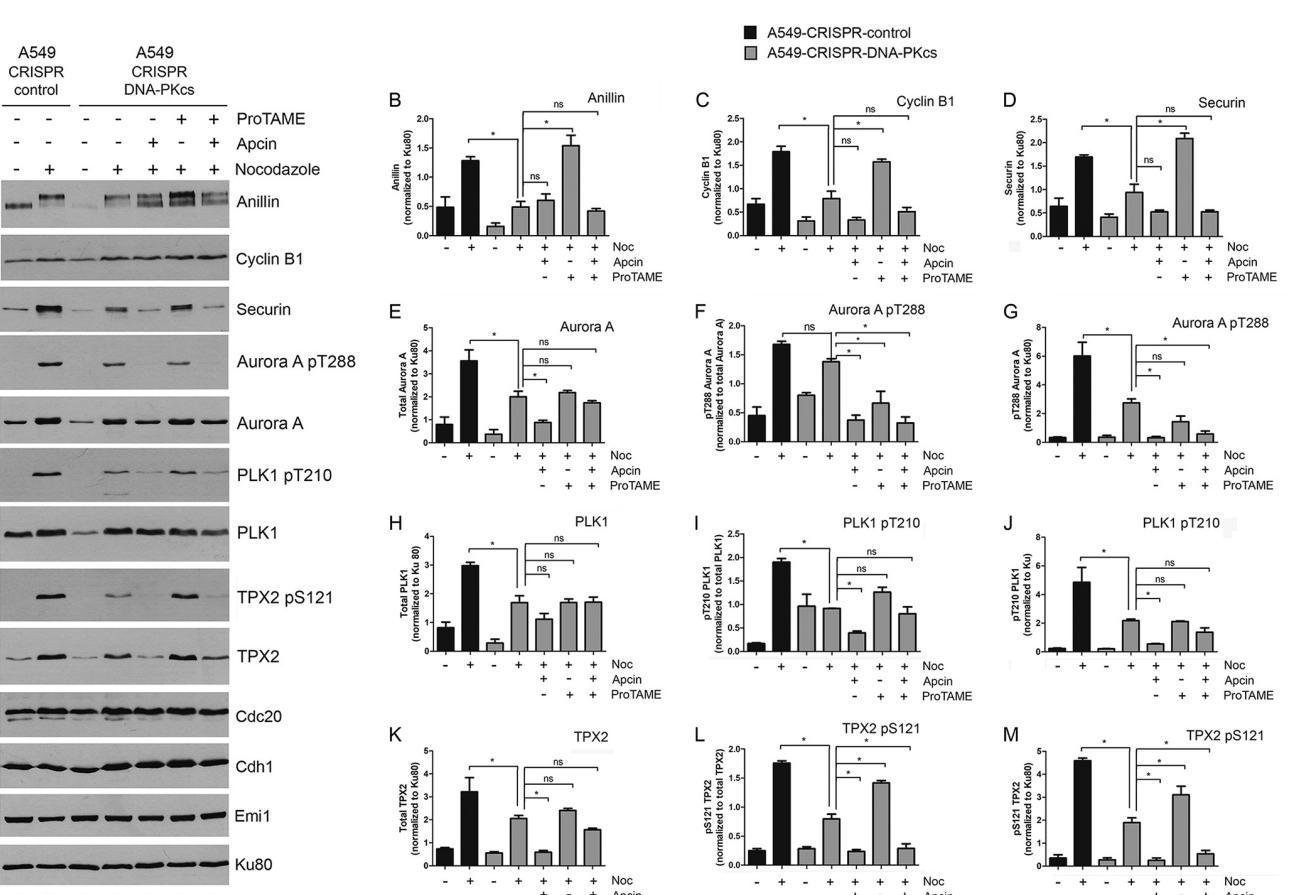
Inhibition of APC/C with proTAME rescues expression and phosphorylation of mitotic proteins in nocodazole-treated DNA-PKcs-deficient A549 cells. Experiments were carried out exactly as for Fig. 7, except that A549-CRISPR-control and CRISPR–DNA-PKcs cells were used. The error bars indicate standard deviations. *, *P* < 0.05; ns, not significant.

DNA-PKcs-deficient cells were preincubated with either apcin or proTAME or both apcin and proTAME for 1 h, followed by addition of nocodazole and incubation for a further 16 h. Remarkably, proTAME, but not apcin, rescued expression of anillin, securin, and cyclin B1, as well as phosphorylation of PLK1, Aurora A, and TPX2, in DNA-PKcs-deficient HeLa and A549 cells (Fig. 7A to D, F, G, I, J, L, and M and 8A to D, F, G, I, J, L, and M), indicating that both the apparent stabilization and phosphorylation of these proteins are regulated in an APC/C-dependent manner in nocodazole-treated cells. We considered that one potential explanation for these results is that loss of DNA-PKcs might decrease the expression of the APC/C activator proteins Cdc20 and Cdh1 or the Cdc20 inhibitor early mitotic inhibitor 1 (Emi1) (49); however, we did not detect a change in expression of these proteins in DNA-PKcs-proficient compared to DNA-PKcs-deficient cells with or without proTAME (Fig. 7 and 8).

## DISCUSSION

DNA-PKcs is a large serine/threonine protein kinase with well-established functions in NHEJ and V(D)J recombination (20, 50). However, recent studies have revealed that its cellular roles are far more extensive than DSB repair alone, ranging from regulation of transcription, metastasis, and cellular senescence to mitosis and cytokinesis (20, 51). Here, we examined the effects of DNA-PKcs loss on entry into mitosis in nocodazole-treated cells. We deleted DNA-PKcs from human cells using CRISPR/Cas9 and showed that these cells have multiple defects in mitosis, including delayed and/or reduced nocodazole-induced upregulation and/or phosphorylation of anillin, securin, cyclin B1, Aurora A, PLK1, and TPX2. Moreover, reduced expression/phosphorylation of these proteins in nocodazole-treated, DNA-PKcs-deficient cells was rescued by inhibition of the APC/C with proTAME, a small molecule that prevents loading of APC/C target-activator complexes, but not apcin, a small molecule that inhibits substrate loading onto Cdc20 and Cdh1.

**FIG 9:**
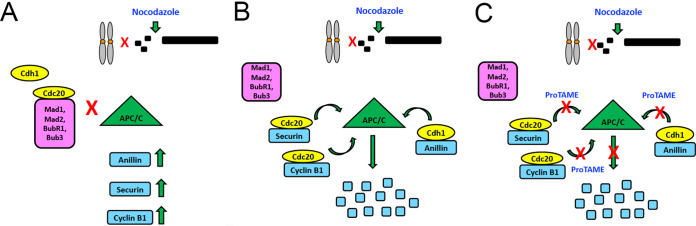
Model for the role of DNA-PK in regulation of APC/C activity in nocodazole-treated cells. (A) Nocodazole causes microtubule depolymerization, preventing attachment of kinetochores to the mitotic spindle. In controls cells, this leads to activation of the SAC; Cdc20 is sequestered by the MCC; APC/C is not activated; and levels of anillin, securin, cyclin B1, and other proteins (not shown) rise. (B) In DNA-PKcs-deficient cells, Cdc20 and Cdh1 interact with and activate the APC/C, causing degradation of mitotic proteins, such as anillin, securin, and cyclin B1, even in the presence of unattached kinetochores. (C) In DNA-PKcs-deficient cells treated with ProTAME, APC/C-mediated degradation of anillin, securin, cyclin B, and other proteins (not shown) is inhibited, causing levels of proteins to rise.

We therefore propose that DNA-PKcs and/or ATM is required for phosphorylation of proteins involved in kinetochore attachment, activation of the SAC, and/or phosphorylation of components of the MCC and/or the APC/C itself (Fig. 9). Although we did not observe changes in the expression of the APC/C activator Cdc20 or Cdh1 or the Cdc20 inhibitor Emi1, changes in phosphorylation cannot be discounted. It is also possible that loss of DNA-PKcs affects interactions between proteins involved in kinetochore attachment, the MCC, and/or the APC/C either directly or in a phosphorylation-dependent manner. Indeed, DNA-PKcs has been reported to interact with the APC/C subunit APC2 and with Cdh1 (18).

It is also possible that the effects of DNA-PKcs loss on the SAC and/or APC/C are a consequence of reduced phosphorylation of CDK1 substrates in G_2_. Indeed, preliminary mass spectrometry experiments revealed that the major effect of loss or inhibition of DNA-PKcs in nocodazole-treated cells was a decrease in phosphorylation in SP and TP sites (data not shown), the known consensus of CDK1 and other proline-directed protein kinases (52, 53). In fact, another study has also proposed that loss of DNA-PKcs delays activation of CDK1 and entry into mitosis (19). Phosphorylation of CDK1 on its inhibitory site, Y15, was increased in nocodazole-treated DNA-PKcs-depleted cells, as were the levels of the protein kinases, Myt1 and Wee1, that add these inhibitory phosphates to CDK1 (19). As shown here, phosphorylation of mitotic proteins recognized by the MPM2 antibody is also decreased, as is the mitotic index of DNA-PKcs-deficient cells (16; data not shown). Thus, it is possible that the multiple mitotic defects in DNA-PKcs-deficient cells could be explained by reduced CDK1/cyclin B1 activity at the G_2_/M transition. Importantly, the decreased levels of cyclin B1 observed here 6 to 16 h after nocodazole treatment could also contribute to reduced CDK1 activity at G_2_/M. Given the importance of CDK1-cyclin B1, not only at entry into mitosis but also in activation of the SAC (54), reduced CDK1 activity could have pleiotropic effects on mitotic outcomes. Our results suggest that the apparent delay in upregulation and phosphorylation of mitotic proteins in nocodazole-treated CRISPR–DNA-PKcs cells is actually due to increased APC/C-mediated proteasomal degradation rather than delayed upregulation. Thus, the decreased cyclin B1 accumulation could represent a feedback mechanism resulting from inability to prevent activation of APC/C.

As discussed above, the APC/C inhibitors apcin and proTAME act in different ways. Apcin prevents interaction of the target protein (for example, cyclin B1 or securin) with Cdc20, while proTAME prevents interaction of the Cdc20 target protein complex with the APC/C (49). Apcin and proTAME are often considered to act synergistically (23); however, in our experiments, we saw clear differences between apcin and proTAME. Addition of apcin had no effect on stabilization of mitotic proteins in nocodazole-treated, DNA-PKcs-depleted cells, whereas addition of proTAME rescued both protein expression and phosphorylation of the mitotic proteins tested. This suggests that proTAME prevents interaction of Cdc20-target protein complexes (cyclin B1, securin, anillin, etc.) with the APC/C, thus preventing degradation of the target proteins. In contrast, when the target proteins are prevented from interacting with Cdc20 by treating the cells with apcin, there may be alternative, APC/C-independent mechanisms for their degradation. Indeed, other E3-ubiquitin ligases, such as Parkin, have been shown to degrade mitotic proteins in an APC/C-independent manner (55). It has previously been observed that even high doses of apcin failed to stabilize some proteins, such as cyclin A2 and Nek2A, whereas proTAME inhibited the degradation of all APC/C substrates tested (23). The authors attributed this difference to the fact that apcin blocks only binding via D box sites, whereas proTAME blocks recruitment of Cdc20 to APC/C (23). Unexpectedly, the combination of apcin and proTAME did not have a synergistic effect on protein rescue in our cells; rather, addition of apcin blocked the ability of proTAME to rescue expression of mitotic proteins, suggesting that inhibition of apcin may upregulate APC/C-independent pathways, thus overriding the effects of proTAME in these cells. In summary, while much work remains to be done, our studies take us a step closer to understanding how loss of DNA-PKcs leads to defects in mitosis in human cells. Given that DNA-PKcs inhibitors are currently in clinical trials (56), determining precisely how DNA-PKcs regulates APC/C-mediated degradation of mitotic proteins could have relevance to cancer therapy.

## MATERIALS AND METHODS

### Reagents and antibodies.

Microcystin-LR was purchased from Enzo Life Sciences. Bovine serum albumin (BSA), phenylmethylsulfonyl fluoride (PMSF), Tris base, EDTA, and nocodazole were purchased from Sigma-Aldrich. Leupeptin, pepstatin, and aprotinin were purchased from Roche. Apcin was purchased from R&D Systems and was resuspended in dimethyl sulfoxide (DMSO) to a 48-mmol/liter stock solution. ProTAME was purchased from Boston Biochem, Inc., as a 20-mmol/liter stock solution in DMSO. MG132 was from Selleck Chemicals. Lambda phosphatase was from New England Biolabs (catalogue number PO753).

Antibodies to actin (no. 3280), anillin (no. 99352), Aurora A pT288 (no. 58494), Cdh1 (118939), Emi1 (no. 187144), histone H2AX (no. 11175), histone H3 (no. 1791), Ku70 (no. 2620), Ku80 (no. 33242), PLK1 (no. 17056), and securin (no. 3305) were purchased from Abcam. Antibodies to ATR (no. 09-070), gamma-H2AX (no. 05-636), histone H3 pS10 (no. 06-570), MPM2 (no. 05368), and mTOR (no. 051564) were purchased from Millipore. The antibody to cyclin B1 was from Santa Cruz (no. 7393), the Aurora A antibodies were from Serotec (no. 2249), PLK1 pT210 was from BD Pharmingen (no. 558400), TPX2 was from Cell Signaling (no. 12245), and Cdc20 (NB100-59828) was from Novus. The antibody to ATM was from Upstate (no. 05-513), and that to PP6 was from Bethyl (A300-844A). Antibodies to DNA-PKcs and TPX2 pS121 were generated in house.

### Cell culture conditions and treatments.

HeLa cells were grown in Dulbecco’s modified Eagle’s medium (DMEM) with 5% serum (HyClone III serum; GE Healthcare), 50 U/ml penicillin, and 50 μg/ml streptomycin in a humidified incubator at 37°C under 5% CO_2_. A549 cells were grown under the same conditions but with 10% serum. Where indicated, nocodazole (Sigma-Aldrich), made up in DMSO, was added to media to a final concentration of 40 ng/ml. Clonogenic survival assays were carried out as described previously (40).

### siRNA.

Smartpool siRNA to PP6c was purchased from Dharmacon and transfected into cells using oligofectamine as described previously (40).

### CRISPR deletion of DNA-PKcs and ATM.

CRISPR deletion of DNA-PKcs and ATM from A549 cells has been described previously (39). For HeLa cells, DNA-PKcs was deleted using the pSpCas9n(BB)-2A-Puro (pX462) vector (Addgene) and short guide sequences targeting exon 35 of DNA-PKcs/*PRKDC* (left, 5′-GCTATAGAAATACTCCCCAT-3′, and right, 5′-GTTCTCAGAAACGATCAACA-3′). All CRISPR deletions were carried out by the Centre for Genome Engineering, Cumming School of Medicine, University of Calgary, Calgary, Alberta, Canada. Additional details are available upon request.

### Cell extracts and Western blotting.

Unless otherwise stated, cells were harvested by resuspension in trypsin EDTA and then washed in phosphate-buffered saline (PBS) and lysed in NETN buffer containing protease inhibitors and microcystin-LR (a phosphatase inhibitor) as described previously (40). For Fig. 5, cells were lysed using Invitrogen cell extraction buffer (catalogue number FN0011) containing protease and phosphatase inhibitors as described above. Protein concentrations were determined using the Bio-Rad detergent-compatible protein assay with BSA as a standard. SDS-PAGE and Western blotting using enhanced chemiluminescence (ECL) reagent and Fuji X-ray film were as described previously (40). Western blots were scanned and quantitated using ImageJ software. Graphs and statistics were developed using GraphPad Prism software. At least 3 separate replicates were analyzed for each experiment. Statistical analysis was carried out using Student's *t* test or one-way analysis of variance (ANOVA), as indicated in the figure legends. *P* values of <0.05 were considered statistically significant.
